# High-Performance Guided Mode Resonance Optofluidic Sensor

**DOI:** 10.3390/s25144386

**Published:** 2025-07-14

**Authors:** Liang Guo, Lei Xu, Liying Liu

**Affiliations:** 1 Key Laboratory for Micro and Nanophotonic Structures (Ministry of Education, China), Department of Optical Science and Engineering, School of Information Science and Engineering, Fudan University, Shanghai 200433, China; 18110720025@fudan.edu.cn (L.G.); lei_xu@fudan.edu.cn (L.X.); 2 Department of Physics, Fudan University, Shanghai 200433, China

**Keywords:** guided mode resonance, optofluidic sensor, optical refractive index sensor, oblique incidence, figure of merit, sensitivity

## Abstract

This paper reports on the high performance of a thick-waveguide guided mode resonance (GMR) sensor. Theoretical calculations revealed that when light incidents on the grating and excites the negative first-order diffraction order, by increasing the waveguide thickness, both a high sensitivity and high figure of merit (FOM) can be obtained. Experimentally, we achieved a sensitivity of 1255.78 nm/RIU, a resonance linewidth of 0.59 nm at the resonance wavelength of 535 nm, an FOM as high as 2128 RIU^−1^, and a detection limit as low as 1.74 × 10^−7^ RIU. To our knowledge, this performance represents the highest comprehensive level for current GMR sensors. Additionally, the use of a microfluidic hemisphere and polymer materials effectively reduces the liquid consumption under oblique incidence and the fabrication cost in practical application. Overall, the proposed GMR sensor exhibits great potential in label-free biosensing.

## 1. Introduction

Since Wood discovered the grating anomaly in 1902 [1], the GMR effect has aroused great interest among researchers. The typical structure of a GMR device is usually composed of four parts: the substrate, waveguide, grating, and cover medium. When under the illumination of a white light source, such structure exhibits a sharp reflection peak and a sharp transmission dip in the spectrum, which originates from the leaky guided modes of the waveguide coupled with the externally propagating diffracted field. Due to its special characteristics, the GMR devices are often used as spectral filters [2,3,4,5], light modulators [6], polarizers [7], broadband mirrors [8,9] and sensors [10,11,12,13,14].

GMR devices were first used as highly sensitive sensors in 1999 [15]. The FOM is an important indicator to evaluate the comprehensive performance of the sensor; it is defined as the ratio of the sensitivity (S) to the full width at half maximum (FWHM) of the resonance curve (FOM = S/FWHM) [16,17]. The FOM of a typical GMR sensor is less than 100 RIU^−1^ [18,19,20,21]. In order to improve the FOM of the sensor, it is necessary to increase sensitivity or reduce FWHM. To increase the sensitivity, Sheng-Fu Lin utilized a metal buffer layer to generate an asymmetric field distribution so that more electric field energy was distributed in the detection region, and the measured sensitivity of the sensor was 376.78 nm/RIU [22]. Ian D. Block [23] and Yuhang Wan [24] both used porous materials with ultralow refractive indexes as the substrates and increased the sensitivity to 450 nm/RIU and 502 nm/RIU, respectively. Min Huang used the free-standing photonic crystals and obtained a sensitivity of 510 nm/RIU [25]. To reduce the FWHM, Yi Zhou proposed a symmetric GMR sensor which can achieve a narrow FWHM [26], while Shuling Wang reduced the FWHM by designing a two-dimensional photonic crystal structure to generate asymmetric Fano resonance [27]. Dmitriin Maksimov changed the incident angle to make the sensors operate in the spectral vicinity of a bound state in the continuum (BIC) to obtain an extremely narrow FWHM [28].

However, it is worth noting that the design of a sensor for obtaining a high FOM needs to be accompanied by a critical design of the measurement apparatus in order to fully exploit the potentialities that a high FOM can offer [29]. Therefore, sensors with both a high FOM and high sensitivity are more desirable in the practical applications [16,17,28,30], because high sensitivity can effectively resist system noise [18,31]. We know that a narrower resonance linewidth means higher confinement of the electromagnetic field energy in the waveguide layer, which results in lower sensitivity [32]. Hitherto, all GMR sensors reported to date have had either high sensitivity or high FOM but not both.

In this paper, we reported a GMR sensor that utilizes the resonance mode excited by the negative first-order diffraction of the grating to achieve both a high FOM and high sensitivity. In our previous work, we demonstrated that the GMR sensor at oblique incidence has a much higher sensitivity than that at normal incidence. When under oblique incidence, the sensor has two resonance modes: positive first-order resonance and negative first-order resonance. For the positive first-order resonance, the total sensitivity is a constructive superposition of grating sensitivity and waveguide sensitivity, and for the negative first-order resonance, the total sensitivity is a destructive superposition of grating sensitivity and waveguide sensitivity [33]. Therefore, a thick waveguide not only provides a lower waveguide sensitivity to maintain the high total sensitivity but can also lead to a narrower resonance linewidth because better electromagnetic field confinement is achieved. By utilizing this feature, we can achieve high sensitivity while obtaining a narrow FWHM, resulting in a high FOM. Experimentally, we achieved a sensitivity of 1255.78 nm/RIU and FOM as high as 2128 RIU^−1^; with that, a detection limit as low as 1.74×10^−7^ RIU is obtained, which represents the highest comprehensive level for current GMR sensors. Our study paves a way for achieving high-performance GMR sensors.

## 2. Theory and Structure Optimization

**Figure 1:**
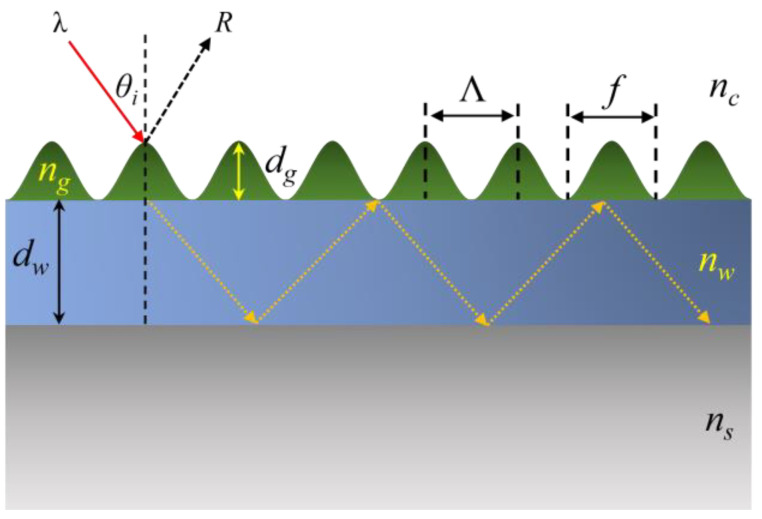
Schematic of a typical GMR with a sinusoidal profile grating. Light incidents (red arrow line) on the grating at *θ_i_*; reflected light (dashed arrow lines) is detected. Yellow dashed arrow lines are resonantly guided light.

Figure 1 is the schematic of a GMR. It consists of a substrate and a cover medium with refractive index *n_s_* and *n_c_*, respectively, a waveguide layer which has a thickness *d_w_* and refractive index *n_w_*, and a grating with a sinusoidal profile; the grating has a depth of *d_g_*, period of Λ, filling factor of *f* and refractive index of *n_g_*.

**Figure 2:**
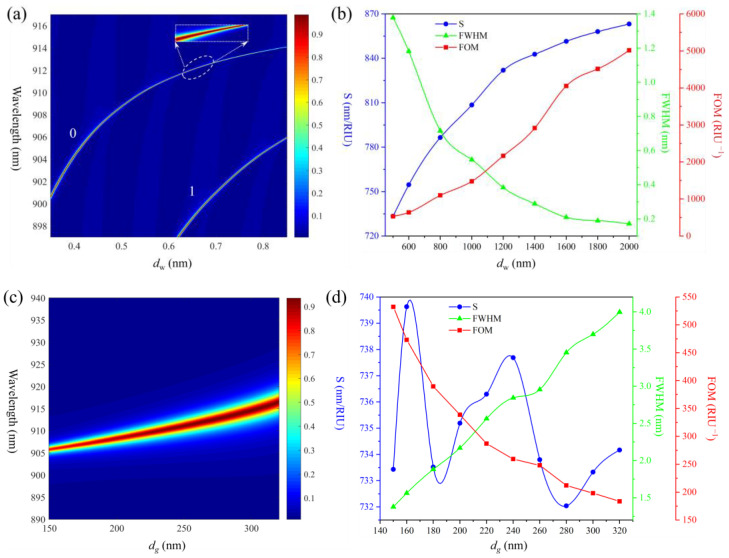

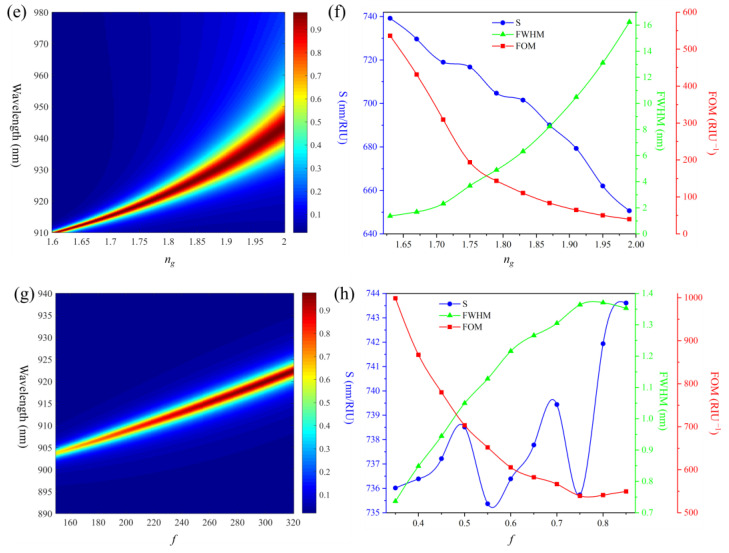
Calculated sensitivity, FWHM, and FOM as a function of (**a**,**b**) different *d_w_*, inset is enlarged area of the white ellipse; (**c**,**d**) different *d_g_*; (**e**,**f**) different *n_g_*; (**g**,**h**) different *f* at *θ_i_* = 40° and (**a**,**c**,**e**,**g**) are the continuous trend of FWHM as a function of the corresponding structural parameters, respectively; the color bar represents the reflectance.

We have found that the sensitivity is directly proportional to the grating period Λ and incident angle *θ_i_* in our previous work. So, in order to achieve higher sensitivity, Λ and *θ_i_* are set to be 1400 nm and 40°, respectively. Additionally, in order to improve the FOM, it is crucial to reduce the sensor’s FWHM. Generally speaking, *d_w_*, *d_g_*, *n_g_* and *f* are the major factors that determine the sensor’s FWHM. We use finite-difference time-domain (FDTD) solutions (Lumerical Inc, Vancouver, Canada.) to calculate the sensor’s FWHM and sensitivity. In this case, *n_w_*, *n_c_*, *n_s_*, *n_g_*, *d_w_*, *d_g_*, and *f* are set to be 1.570, 1.333, 1.450, 1.630, 500 nm, 150 nm, and 0.8, respectively; the calculated FWHM, sensitivity and FOM versus *d_w_*, *d_g_*, *n_g_* and *f* are shown in Figure 2.

Figure 2a shows the calculated FWHM as a function of *d_w_* (500~2000 nm). The color bar represents the reflectance, which can describe the FWHM of the resonance wavelength, ‘0’ represents the fundamental mode of the waveguide, and ‘1’ represents the first-order mode of the waveguide (higher-order waveguide modes will generate as the *d_w_* increases; here, we only consider the fundamental mode of the waveguide). It is clear that a thicker waveguide corresponds to a narrower FWHM. Figure 2b shows the calculated sensitivity and FOM versus *d_w_*; as observed in the figure, a thicker waveguide corresponds to higher sensitivity. As a result, the FOM grows from about 500 at *d_w_* = 600 nm to about 5000 at *d_w_* = 2000 nm. Figure 2c,d show the calculated performance versus *d_g_* (150~320 nm). We can learn that the increased *d_g_* broadened the FWHM while the sensitivity does not exhibit a clear pattern of change, and the FOM decreases as the *d_g_* increases. Figure 2e,f show the relationship between *n_g_* (1.6~2.0) and the sensor’s performance. It can be seen that a smaller *n_g_* corresponds to a narrower FWHM and higher sensitivity, which is the same as a thicker waveguide. Figure 2g,h show the sensor’s performance versus *f* (0.35~0.85); the change rules are similar to those of Figure 2c,d.

We can find that increasing *d_w_* and reducing *n_g_* can both reduce the FWHM while improving sensitivity. This is because the increased *d_w_* and reduced *n_g_* allow more electromagnetic field energy to be localized in the waveguide layer with less leakage, which results in a narrower FWHM and lower waveguide sensitivity. Meanwhile, reducing *d_g_* and *f* means that the effective refractive index of the grating layer decreases, which is equivalent to reducing *n_g_* and thus also results in a narrower FWHM. However, reducing *d_g_* and *f* leads to structural changes that alter the distribution of the electromagnetic field, thereby affecting the sensitivity and ultimately resulting in a trade-off between sensitivity and FOM. Additionally, it is observed that *d_w_* exerts the most significant influence on the FOM, whereas the impacts of *d_g_*, *n_g_* and *f* on the FOM are relatively minor.

Therefore, a thicker waveguide is the most straightforward way to achieve both high sensitivity and FOM. Moreover, reducing *n_g_* is also a viable strategy, as it can improve the sensitivity and FOM simultaneously. *d_g_* and *f* affect the sensitivity, so we do not need to deliberately optimize them; this can also reduce the difficulty of sensor fabrication.

## 3. Fabrication of GMR Optofluidic Sensor

**Figure 3:**
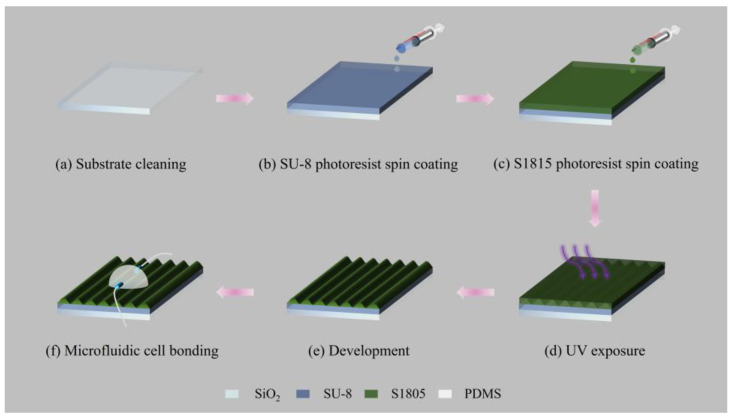
Diagram for the fabrication flow of optofluidic GMR sensor. (**a**) Clean the SiO_2_ substrate; (**b**) spin coating the SU-8 photoresist on the substrate; (**c**) spin coating the S1815 photoresist on the waveguide; (**d**) UV exposure; (**e**) development; (**f**) bond the PDMS microfluidic hemisphere to the GMR chip.

Figure 3 shows the fabrication flow chart to fabricate the GMR sensor. An SU-8 photoresist is used as the waveguide material, and an S1815 photoresist is used as the grating material. The cheap polymeric material is the lowest possible cost price of the sensor, which is competitive with those produced using expensive vacuum-based methods.

The SiO_2_ substrate is cleaned by rinsing with acetone and alcohol for 3 min respectively in an ultrasonic cleaner, which is followed by washed with deionized water and then drying in an oven at 80 °C for 2 min (Figure 3a). After that, a 900 nm thick SU-8 2000.5 photoresist is spin coated on the substrate, exposed to UV light for 30 s, and then hard baked at 150 °C for 2 h (Figure 3b). A ~300 nm thickness S1815 photoresist layer is subsequently spin-coated on the SU-8 waveguide layer; the grating pattern is formed by exposing with a helium–cadmium laser (Figure 3c,d) and developing with 2.38% tetramethylammonium hydroxide (NMD-3) solution (Figure 3e). Finally, the hemisphere shell of polydimethylsiloxane (PDMS) is bonded to the GMR chip by UV glue (Norland, NOA61), as shown in Figure 3f.

**Figure 4:**
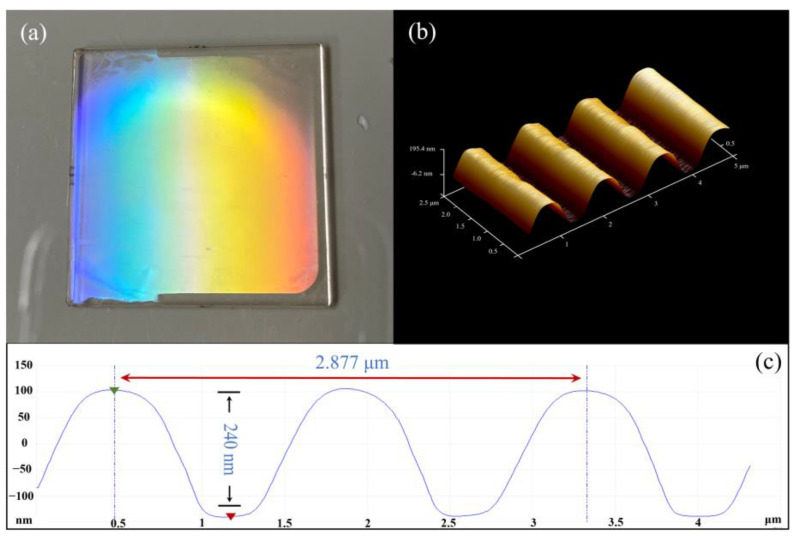
(**a**) Photograph of the manufactured GMR sensor; (**b**) 3D image of the holographic grating pattern obtained by AFM; (**c**) profile of the holographic grating scanned by AFM, the green triangle represents the highest point, and the red triangle represents the lowest point; the measured grating period is 1438.5 nm, and the grating depth is 240 nm.

Figure 4a shows the photo of the fabricated GMR. Figure 4b,c show the grating profile measured by an atomic force microscope (AFM, Bruker (Billerica, MA, USA), Dimension 5000). The depth of the grating is 240 nm, and the average period is 1438.5 nm. The grating has a sinusoidal profile with a filling factor of about 0.8.

**Figure 5:**
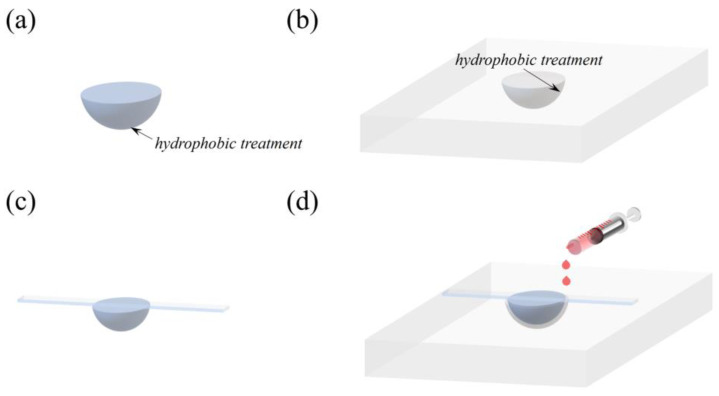
Diagram for the fabrication flow of the homemade PDMS hollow hemisphere. (**a**) SiO_2_ hemisphere; (**b**) hollow silicone mold; (**c**) paste a glass strip onto the SiO_2_ hemisphere; (**d**) place the SiO_2_ hemisphere into the silicone hemisphere, and inject the PDMS into the voids.

The microfluidic hemisphere is fabricated by embedding a SiO_2_ hemisphere (shows in Figure 5a) into a hollow silicone mold (shows in Figure 5b), and the fabrication flow is shown in Figure 5. First, the SiO_2_ hemisphere is washed with acetone and alcohol for 2 min, respectively, in an ultrasonic cleaner, followed by washed with deionized water, and then dried in an oven at 80 °C for 2 min. Subsequently, the outer surface of the SiO_2_ hemisphere is hydrophobized with trichloromethylsilane (CH_3_SiCl_3_), which makes the PDMS hemisphere easier to peel off and ensures the inner surface is smooth enough to reduce light scattering (Figure 5a).

Then, the same hydrophobic treatment is applied to the inner surface of the silicon mold, which can make the outer surface of the PDMS hemisphere smooth (Figure 5b). After that, a glass strip is applied onto the SiO_2_ hemisphere, as shown in Figure 5c, and it is placed into the silicone hemisphere, as shown in Figure 5d. After adjusting the position of the SiO_2_ hemisphere so that the center of the SiO_2_ hemisphere coincides with the center of the hemisphere mold, the glass strip is fixed on the silicone mold. Then, the PDMS prepolymer (its curing agent was mixed in a mass ratio of 5:1) is injected into the voids between the SiO_2_ hemisphere and the hollow hemisphere mold. Paying attention to the changes in the voids during the injection process, we reduced the injection rate when the voids were about to be filled immediately and then transferred the mold into a vacuum box to remove the bubbles from the PDMS. After that, we cured the PDMS prepolymer at 80 °C for 4 h and then peeled off the PDMS microfluidic hemisphere from the SiO_2_ hemisphere and silicon mold gently.

**Figure 6:**
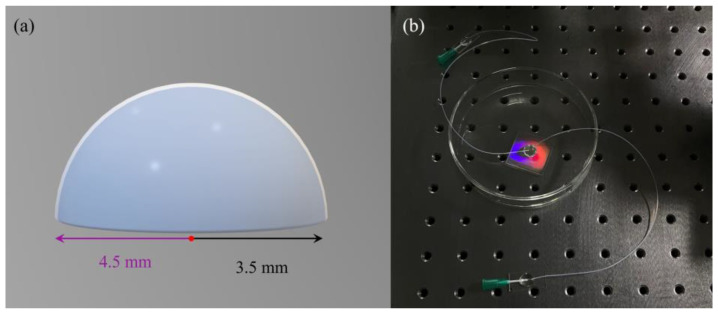
(**a**) Schematic of the PDMS hollow hemisphere; (**b**) photograph of the fabricated GMR optofluidic sensor.

Figure 6a shows the schematic of the homemade PDMS hollow hemisphere, which ensures that the incident light irradiates on the surface of grating at any angle. The PDMS hollow hemisphere has an outer radius of 4.5 mm and inner radius of 3.5 mm (if the hemisphere dimensions are too small, it cannot connect microfluidic pipes at both ends; after several attempts, we finally chose these dimensions), while its inner volume is ~89 μL; this structure can effectively solve the problem of large liquid consumption under oblique incidence in previous studies [33,34]. Finally, we connected two microfluidic pipes to the hemisphere acting as the inlet and outlet ends (Figure 6b shows the photo of the manufactured GMR optofluidic sensor).

## 4. Experimental Results and Discussion

**Figure 7:**
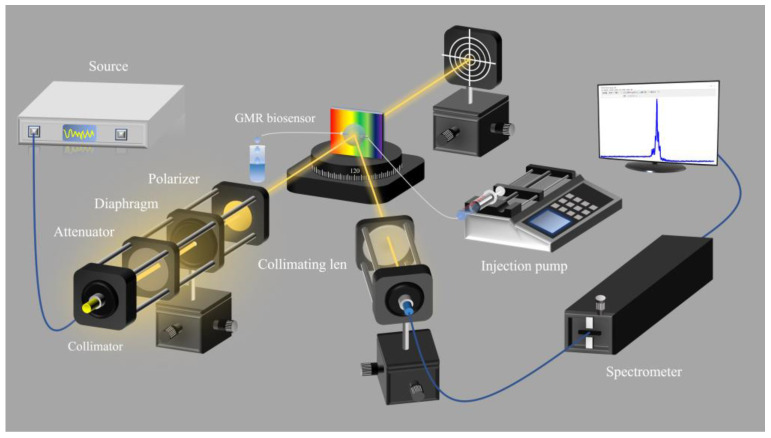
Schematic illustration of the experimental optical detection system for measuring the reflectance spectra of the GMR optofluidic sensor.

The experimental setup for refractive index change measurement is shown in Figure 7. Light emitted from a super-continuous light source (YSL Photonics, SC-5, spectral range: 470–2400 nm) was guided along a fiber collimator to form a collimated light beam. The collimated light beam passed through an attenuator, a diaphragm (the diameter of the spot is 0.8 mm; this size can effectively reduce the influence of fabrication imperfections on sensor performance) and a polarizer; it then irradiated on the GMR optofluidic sensor. The reflected light beam was collected by a spectrometer (Acton Research (Acton, MA, USA), SpectraPro-2750).

**Figure 8:**
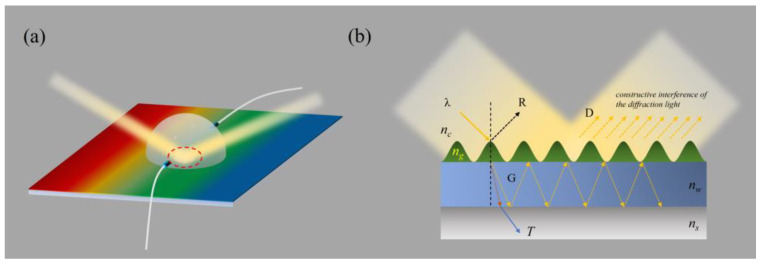
(**a**) The propagation path of light entering the PDMS hemisphere; (**b**) the propagation path of light entering the sensor; the enlarged image of the red circle area is shown in (**a**).

We made a mark at the center of the PDMS hemisphere module on the back of the sensor, and the incident light irradiated on the center of the sphere. This step can ensure that the incident light is perpendicular to the surface of the PDMS hemisphere module, as shown in Figure 8a. Figure 8b shows the propagation path of light entering the sensor (the enlarged red circle area in Figure 8a). The light of wavelength λ is incident on the sensor, and part of the light undergoes reflection (R) and transmission (T), while the remaining light is coupled into the waveguide to form guided modes (G). Due to the diffraction of the grating, the guided modes will leak during propagation. The leaked guided modes (D) interfere with the external scattering field, forming a strong reflection peak, which is the detected resonance peak.

We characterized the sensing performance of the GMR sensor by taking refractive index measurements and measuring the reflection spectra at different concentrations of aqueous glycerol solutions. The concentration of glycerol was in a range of 0–16% by volume, and the corresponding refractive index was 1.333–1.3547. The incident angle was set at *θ_i_* = 40°, 50°, 60° and 65°, respectively. Liquid was injected into the hollow hemisphere by an injection pump. The microfluidic channel was washed 3 times with deionized water for the next solution measurement.

**Figure 9:**
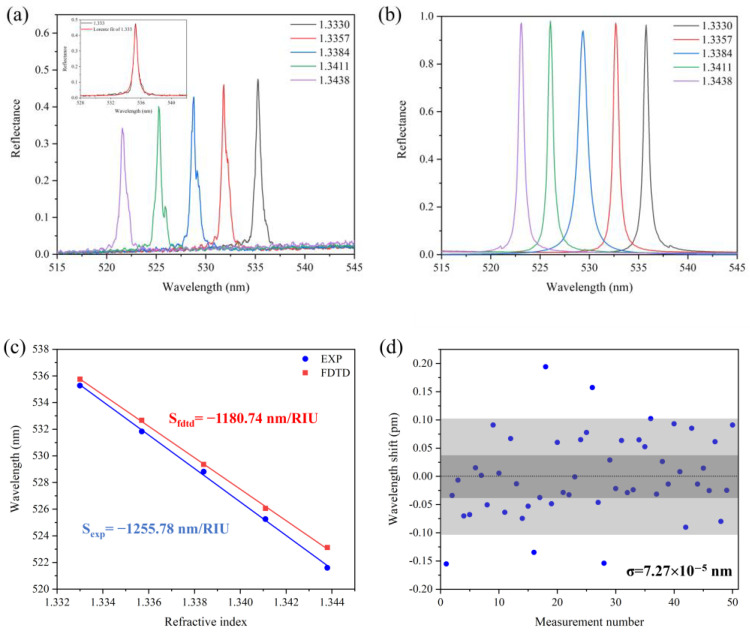
TM mode reflectance spectra of the GMR sensor at incident angle *θ_i_* = 65°. (**a**) Experimental results; (**b**) simulation results; (**c**) fitted sensitivity; (**d**) resonance wavelength from 50 measurements, the gray area represents the 3σ deviation.

Figure 9a–c show the experimental and simulated TM mode reflectance spectra of the sensor at *θ_i_* = 65°. A linear fitting gives a sensitivity of 1255.78 nm/RIU. In comparison, the simulated sensitivity is 1180.74 nm/RIU, which is in good agreement with the experimental result.

Table 1 summarizes the experimental and simulated sensitivity, linewidth and FOM; the linewidth is obtained by Lorentzian fitting of the resonance.

It can be seen from the table that due to the characteristic of the 1st negative resonance, as the incident angle increases, the resonance wavelength shifts from the near-infrared band to the visible light band, which is very friendly for applications related to biosensing. The measured linewidths are all around 1 nm, which leads to a high FOM. In addition, the linewidth of the TM mode is narrower than that of the TE mode, because the TM mode has a lower transmission loss, and the electromagnetic field energy can be better confined in the waveguide layer. The sensitivity of the TE mode is higher than that of the TM mode. The highest sensitivity appears at an incident angle of 65°. As far as we know, 1255.78 nm/RIU is the highest value for GMR sensitivity, which is a 6~12-fold improvement compared to other reported GMR sensors.

Table 2 lists the performances of the reported GMR sensors. The FOM of our work is at least one order of magnitude higher; meanwhile, our GMR sensitivity is also several times higher.

The detection limit (DL) is a key parameter in practical applications, which is defined as 3σ/S [18,31], in which σ is the standard deviation of the resonance peak wavelength obtained from multiple measurements. We measured 50 sets of resonance curves with a time interval of 1 min when the sensor was immersed in deionized water, and the distribution of the center wavelength relative to the mean wavelength is shown in Figure 9d. The resonance wavelength follows a normal distribution with σ = 7.27 × 10^−5^ nm, which indicates that the sensor has good wavelength stability and repeatability. This yields a low detection limit of 1.74 × 10^−7^ RIU, which is superior to the existing reported values [26,27,38].

The factors that affect the long-term stability and repeatability of the sensor usually include temperature, humidity, system noise, vibration, fabrication imperfections, etc. So, the long-term stability and repeatability of the sensor varies under different conditions. We have conducted wavelength stability and repeatability testing with a testing period of 4 h. During this period, we recorded the resonance wavelength every minute, and the drift of the resonance wavelength was in the order of pm, and mainly caused by system noise, demonstrating good wavelength stability and reproducibility.

We also evaluated the impact of temperature changes and fabrication imperfections on the sensor’s stability and repeatability. Temperature changes can affect the refractive index of materials; for this sensor, the refractive index of the cover medium (*n_c_*) has the greatest impact on the resonance wavelength and sensitivity. If the temperature changes by 0.1 °C, *Δn_c_*≈0.000009, in this situation, the resonance wavelength shifts by 0.01 nm and the sensitivity changes by 0.0013 nm/RIU. We can see that temperature changes have a minimal impact on the resonance wavelength and sensitivity.

For the fabrication imperfections, we mainly consider the grating period and waveguide thickness. The non-uniformity of the grating period is usually less than 1 nm; if the grating period changes from 1438.5 nm to 1439.5 nm, the resonance wavelength changes from 535.75 nm to 536.07 nm, the sensitivity changes from 1180.74 nm/RIU to 1181.49 nm/RIU. The non-uniformity of the waveguide thickness is usually less than 10 nm; if the waveguide thickness changes from 900 nm to 910 nm, the resonance wavelength changes from 535.75 nm to 536.12 nm, the sensitivity changes from 1180.74 nm/RIU to 1182.10 nm/RIU. It can be seen from the calculation results that the fabrication imperfections have a relatively small impact on the performance of the sensor. We minimized the size of the incident light spot as much as possible while keeping the incident area unchanged as a feasible solution to this problem.

The SPR detection system by Biacore is usually considered as the gold standard in the biosensor landscape, and it is usually used as a reference to evaluate the performances of other sensors [29]. The advantage of SPR sensors is their higher sensitivity compared to dielectric ones, usually above 1000 nm/RIU, while the GMR sensors usually have the values of 100~200 nm/RIU. However, SPR sensors have wider FWHM values due to the lossy nature of the metal, which significantly decreases the FOM. Therefore, typical GMR sensors usually have a similar magnitude of FOM compared to SPR sensors, but the lower sensitivity of the GMR sensors still limits their development, although they are easy to interface. Therefore, a horizontal comparison was taken with the SPR sensors, and Table 3 lists properties of different types of typical SPR sensors as references to our GMR sensor. The high GMR sensitivity in this work is comparable to most of the existing reported grating-coupled SPR sensors [39,40], nanoparticle (NP)-based LSPR sensors [41,42,43] and nanohole (NH)-based LSPR sensors [44,45,46]. On the other hand, the ultra-high FOM in this work exceeds almost all SPR sensors, including BIC-type SPR sensors [47] and hybrid-type SPR sensors [48,49,50], which aim to reduce the FWHM of the resonance curve. Note that reducing the linewidth requires a much more sophisticated fabrication process. To the contrary, the GMR sensor in this work has the advantage of a relatively simple fabrication process and low fabrication cost.

In addition, we compare the performance of our GMR sensor with the sensors listed in this review article, which focus on optical refractive index sensors [16], including dielectric-based propagating photonic eigenwave sensors, dielectric-based localized photonic eigenmode sensors, 2D material integrated sensors, etc., and the results show that our GMR sensor has excellent performance in the field of optical refractive index sensing.

The performance of the GMR sensor can be further improved by using a larger grating period and a thicker waveguide layer. Taking Λ = 2000 nm, *d_w_* = 2000 nm, *d_g_* = 150 nm, *θ_i_* = 65°, *f* = 0.5, and leaving other parameters remain unchanged, the FDTD calculation indicates that the sensitivity can reach up to 1738.48 nm/RIU at 729 nm, while the linewidth drops to 0.099 nm, leading to an ultra-high FOM of 17,560 RIU^−1^.

## 5. Conclusions

In summary, we reported a new type of GMR optofluidic sensor with a thick 900 nm SU-8 waveguide layer. When light incidents at 65° and the 1st negative diffraction mode is excited, the sensor has a sensitivity of 1255.78 nm/RIU and an FOM of 2128 RIU^−1^, while its detection limit is as low as 4.6 × 10^−7^ RIU. We also predict that the FOM can go as high as 17,560 RIU^−1^ with proper design of the GMR structure, combining the characteristics of visible light band response and low fabrication cost, making the GMR a very challenging sensor scheme to compete with standard techniques such as the SPR technique.

## Figures and Tables

**Table 1:** Experimental and simulated results of sensitivity, linewidth, and FOM under different incident angles and polarization modes.

	Experimental Results			Simulation Results		
Implementation	S (nm/RIU)	FWHM (nm)	FOM (RIU^−1^)	S (nm/RIU)	FWHM (nm)	FOM (RIU^−1^)
TE-1 40°	922.55	1.48	643.61	823.63	1.24	664.22
TM-1 40°	912.69	1.26	724.36	802.45	0.63	1273.73
TE-1 50°	1099.84	1.70	646.96	1008.25	1.39	725.36
TM-1 50°	1093.55	1.61	679.22	1003.77	0.80	1254.71
TE-1 60°	1221.14	1.29	946.62	1154.48	1.04	1110.08
TM-1 60°	1202.48	0.81	1484.54	1133.81	0.69	1643.20
TM-1 65°	1255.78	0.59	2128.44	1180.74	0.62	1904.42

**Table 2:** Comparison of the resonance wavelength, sensitivity, linewidth, FOM, and detection limit of different structures and fabrication techniques of GMR sensors.

Structures/Implementations	λ_R_ (nm)	S (nm/RIU)	FWHM (nm)	FOM (RIU^−1^)	DL (RIU)	Ref.
Typical structure	~774	87.88	~7	~13	3.4 × 10^−5^	[10]
All-polymer	573	31	0.9	34	4.5 × 10^−6^	[18]
Oblique-angle layer deposition	~597	61	~6	~10	/	[19]
Chirped grating period	~676	160	12	13	7.0 × 10^−6^	[20]
Low-index cavity layers	~637	181.9	~9.2	~20	/	[21]
Metal layer assisted	~743	376.78	~15	~25	/	[22]
Low-index porous substrate	833	300	3.5	~86		[23]
Ultralow RI substrate	~798	502	~10	~50	/	[24]
Free-standing photonic crystals	~850	510	~10	~51	/	[25]
Oblique incidence condition	807	452.1	~5	~90	/	[34]
Agarose-Gel Based	848	450	~5	~90	/	[35]
Waveguide grating imager	828.8	113.85	1.593	71	2.2 × 10^−6^	[36]
Single-layer	616	229.43	7.28	32	/	[37]
This work	535	1255.78	0.59	2128	1.74 × 10^−7^	

**Table 3:** A comparison of performances between our GMR sensor with different types of SPR sensors.

Types	λ_R_ (nm)	S (nm/RIU)	FOM (RIU^−1^)	Ref.
Grating coupling	1060	859.2	62.5	[39]
Grating coupling	1020	858	150.46	[40]
Metallic NP-based LSPR	1036	770	2.42	[41]
Metallic NP-based LSPR	1545	880	2	[42]
Metallic NP-based LSPR	786	540	21.5	[43]
Nanohole-based LSPR	985.5	753.06	2091	[44]
Nanohole-based LSPR	~1547	1022	410	[45]
Nanohole-based LSPR	~1541	1050	208	[46]
BIC in plasmonic	~874	657	109	[47]
Hybrid types SPR	1288	1015	108	[48]
Hybrid types SPR	1318	926	252	[49]
Hybrid types SPR	824	717	162.2	[50]
Prism coupling	510	2875	121.31	[51]
Prism coupling	693	4436	75.19	[52]
Waveguide coupling	1478.2	38978	159.87	[53]
This GMR sensor	535	1255.78	2128	

## Data Availability

The data that support the findings of this study are available from the corresponding author upon reasonable request.
